# Do Serum Nesfatin-1 Levels have A Predictive Role in Type-2 Diabetes Mellitus and its Microvascular Complications? A Case-Control Study

**DOI:** 10.7759/cureus.53007

**Published:** 2024-01-26

**Authors:** Usama A Khalil, Osama E Mohamed, Abdullah A Abdullah, Mohamed S Fawzy, Nearmeen M Rashad, Ghada M Samir

**Affiliations:** 1 Internal Medicine, Faculty of Medicine, Zagazig University, Zagazig, EGY; 2 Medical Research Group of Egypt, Negida Academy, Arlington, MA, USA; 3 Clinical Biochemistry, Faculty of Medicine, Zagazig University, Zagazig, EGY

**Keywords:** insulin, glucose, kidney, type-2 diabetes mellitus, nesfatin-1

## Abstract

Background

Type 2 diabetes mellitus (T2DM) is a chronic disease with macrovascular and microvascular complications. Nesfatin-1 is a neuropeptide that develops from a more substantial intermediate compound known as nucleobindin 2 (NUCB2). Nesfatin-1 is known to play a role in regulating various physiological processes related to appetite, energy balance, and body weight. The purpose of the current study was to investigate the serum levels of nesfatin-1 in Egyptian patients with type 2 diabetes mellitus (T2DM) in comparison to healthy subjects and to assess the association of serum nesfatin-1 levels with the occurrence of diabetic microvascular complications in those patients.

Methods

This matched case-control study was conducted on 90 subjects 40-80 years old, with normal hepatic, cardiac, and respiratory functions, and 60 of them had T2DM. The included participants were divided into two groups: group 1, which was the control group and included 30 healthy subjects, and group 2, which included 60 subjects with T2DM. Group 2 was subdivided according to the presence or absence of microvascular complications into group 2a, which included 30 patients having T2DM with no microvascular complications, and group 2b, which included 30 patients having T2DM with one or more microvascular complications.

Results

T2DM patients had significantly lower serum nesfatin-1 levels (5.07±1.78 versus 9.05±2.1 mmol/L, <0.001) compared to healthy controls. Also, T2DM patients with microvascular complications had lower serum nesfatin-1 levels (4.32±1.72 versus 5.83±1.51 mmol/L, <0.001) compared to T2DM patients without microvascular complications. Serum nesfatin-1 level at a cutoff value of <8.09 mmol/L can be a marker for the detection of diabetes mellitus (DM) with the area under the curve (AUC) of 94.3%, 95% sensitivity, 74.3% specificity, 77.9% positive predictive value (PPV), and 65.7% negative predictive value (NPV), and at a cutoff value of <5.87 mmol/L can be a marker for the detection of microvascular complications of diabetes mellitus at AUC of 75.5%, 76.7% sensitivity, 67.3% specificity, 77.1% PPV, and 62.9% NPV.

Conclusions

Serum Nesfatin-1 may play a potential protective role in diabetes mellitus (DM) and its microvascular complications, as it decreases in individuals with diabetes and those with diabetic microvascular complications compared to controls. Additionally, serum Nesfatin-1 levels may have predictive value for the early detection of Type 2 diabetes mellitus (T2DM) patients, diabetic microvascular complications, and diabetic kidney disease (DKD) at cut-off values of < 8.09 (mmol/L), < 5.87 (mmol/L), and < 5.46 (mmol/L), respectively. Therefore, targeted Nesfatin-1 drug therapy may be tried to reduce morbidity and mortality caused by microvascular complications of diabetes.

## Introduction

Hyperglycemia is a symptom of the persistent, progressing condition known as diabetes mellitus (DM). It is an important medical condition that is becoming more prevalent all over the world. Around 463 million people worldwide have diabetes at this time, and one in 11 adults (20-79 years old) has the disease on average. According to data from the Centers for Disease Control and Prevention Diabetes Surveillance System, approximately 11.3% of adults had a diagnosis of diabetes in 2022, 37.3 million people, including 28.7 million with a diagnosis and 8.5 million undiagnosed, with 95% of them being with type 2 diabetes mellitus (T2DM) [1,2].

The chronic effects of diabetes have an influence on nearly every organ in the body, but especially, significant organs such as the kidney, heart, and brain are affected. Vascular concerns, caused by abnormal micro- and macrovascular mechanisms and operations, are the main cause of multiorgan failure and increased death and morbidity from diabetes-related complications [2].

Diabetic nephropathy, diabetic retinopathy, and diabetic neuropathy are examples of microvascular problems. Among the severe microvascular consequences of diabetes is diabetic nephropathy. Both in industrialized and developing nations, it is the primary contributor to renal failure and the greatest killer of people with diabetes. More than 3.48 million people died from diabetic nephropathy in 2017, a 145% increase from 1990 [3]. In industrialized nations, diabetic retinopathy is the most common cause of visual loss in adults with jobs (20-65 years old) [4]. Diabetic microvascular problems have been found to benefit from strict glucose control [5].

A neuropeptide called nesfatin-1 was originally identified in 2006. It develops from a more substantial intermediate compound known as nucleobindin 2 (NUCB2). The brain, notably the hypothalamus, brainstem, and pituitary gland, as well as the adipose tissue, stomach, and pancreas, are the primary sites of nesfatin-1 expression [6].

It is well recognized that nesfatin-1 regulates a number of bodily functions that affect hunger, energy expenditure, and weight distribution. It has been discovered to have anorexigenic properties, which means that it can reduce appetite and increase satiety. Additionally, it controls lipid metabolism, insulin secretion, and the balance of glucose. It has been demonstrated that nesfatin-1 increases the responsiveness to insulin and encourages pancreatic beta cells to secrete more insulin [7].

In the condition of a high level of glucose, nesfatin-1 was observed to increase cell viability and protect ARPE-19 cells from inflammation, oxidative stress, and apoptosis brought on by elevated glucose levels. Nesfatin-1 also suppressed the expression of HMGB1 and the activation of the nuclear factor kappa B (NF-κB)/NLRP3 inflammasome pathway, thus inhibiting their inflammatory actions [8].

To our knowledge, many studies have been done in this field investigating serum Nesfatin-1 in T2DM and prediabetic patients, but a few have been done in diabetic patients with microvascular complications with conflicting results, so we designed this study to measure the serum levels of Nesfatin-1 in Egyptian patients with T2DM in comparison to normal populations and to determine whether these differences were associated with the development of diabetic microvascular events in those patients and the determination of Nesfatin-1 level cut-off values for detection of DM and its microvascular complications, especially DKD.

## Materials and methods

Study design and setting

At the Internal Medicine and Clinical Biochemistry Departments, Faculty of Medicine, Zagazig University Hospitals, from January 2022 to August 2023, 90 subjects between the ages of 40 and 80 with normal hepatic, cardiac, and respiratory functions participated in this matched case-control study, 60 of whom had T2DM.

The subjects gave their written informed consent to take part in the investigation. After receiving Institutional Review Board (IRB) approval, the Internal Medicine and Clinical Biochemistry Departments at Zagazig University Hospitals gave their consent for the study to be conducted (ZU-IRB #9094/16-11-2021).

Inclusion and exclusion criteria

We included patients aged 40-80 years of either sex, healthy controls, and individuals with T2DM with and without microvascular complications. We excluded patients with liver or kidney dysfunction other than DKD, chronic inflammatory diseases, untreated hypertension, autoimmune disorders and malignancies, and any other endocrine diseases except T2DM.

The included participants were divided into two groups: group 1, which was the control group and included 30 healthy subjects, 13 of whom were males and the other 17 subjects were females, and group 2, which was the T2DM group and included 60 T2DM patients who were diagnosed according to the ADA criteria (2022) [9], 27 of whom were males and 33 were females; this group was sub-divided according to the presence or absence of microvascular complications into group 2a, which included 30 patients having T2DM with no microvascular complications, and group 2b, which included 30 patients having T2DM with one or more of microvascular complications such as clinical peripheral neuropathy (diagnosed by history-taking; Douleur Neuropathique 4 Questions {DN4} questionnaire scores of ≥4/10, which indicates neuropathic pain; and neurological examination) retinopathy (diagnosed by fundus examination), and nephropathy (diagnosed by urine albumin-creatinine ratio {UACR}).

Clinical assessment and outcomes

The following tests were applied to each study participant: complete history and comprehensive clinical evaluation including the measurement of blood pressure; body mass index (BMI) calculation; the measurement of waist circumference using a tape measure; pinprick test for clinical peripheral neuropathy diagnosis; regular laboratory tests such as urine analysis and whole blood count; blood coagulation tests including prothrombin time (PT), partial thromboplastin time (PTT), international normalized ratio (INR), glycated hemoglobin (HbA1c), two-hour postprandial plasma glucose, and fasting plasma glucose; renal function tests including serum creatinine, serum urea, creatinine clearance, and the estimation of estimated glomerular filtration rate (eGFR); UACR; and liver function tests including serum alanine transaminase (ALT) and aspartate aminotransferase (AST), serum albumin, and serum bilirubin (total and direct) and other routine investigations including electrocardiogram (ECG) and abdominal ultrasound, fundus examination for the assessment of retinopathy, the measurement of fasting insulin (μIU/mL), the measurement of insulin resistance by homeostasis model assessment (HOMA-IR) index by using computer analyses (i.e., fasting blood glucose and insulin levels); DN4 questionnaire for the assessment of neuropathic pain, and serum nesfatin-1 level measurement using the enzyme-linked immunosorbent assay (ELISA) technique (mmol/L).

Sample size calculation

Using the OpenEpi software [10], we calculated the sample size for cases and controls. Based on a previous study [11], assuming the mean nestafin-1 level was 1.09±0.1 versus 1.039±0.09 ng/mL in control versus cases, at 80% power and 95% CI, the estimated sample was calculated to be 90 subjects, with 30 subjects in each group.

Statistical analysis

Statistical Package for Social Sciences (SPSS) v25 (IBM SPSS Statistics, Armonk, NY) was used for the statistical analysis. The mean and standard deviation (SD) of the quantitative data were reported, and Student's t-test was used to compare them. The frequency and percentage of the qualitative characteristics were shown. To determine how closely two variables correlate with one another, the correlation coefficient (r) was determined. The association was deemed weak if r was between 0.1 and 0.3, moderate if it was between 0.3 and 0.6, and strong if it was over 0.6. Using the receiver operating characteristic (ROC) curve, the diagnostic performance's area under the curve (AUC), sensitivity, specificity, positive predictive value (PPV), and negative predictive value (NPV) were assessed.

## Results

Comparison between type 2 diabetes patients and healthy controls

T2DM patients were associated with statistically significant higher BMI, waist circumference, systolic and diastolic blood pressure, UACR, total cholesterol (TC), triglycerides (TG), low-density lipoprotein (LDL) cholesterol, fasting plasma glucose (155.7±62.9 versus 88.6±7.8 mg/dL), postprandial plasma glucose (260.33±56 versus 135±22.36 mg/dL), HbA1c (8.3±1.5 versus 4.84±0.49), fasting serum insulin (24.6±13.92 versus 6.87±1.91 µIU/mL), and HOMA-IR (8.15±5.18 versus 1.4±0.438), while they had statistically significant lower eGFR, high-density lipoprotein (HDL) cholesterol, and serum nesfatin-1 level (5.07±1.78 versus 9.05±2.1 mmol/L) compared to healthy controls (Table 1).

**Table 1:** Comparison between diabetes patients and the control group according to baseline characteristics and laboratory investigations. *Statistically significant BMI, body mass index; AST, aspartate aminotransferase; ALT, alanine transaminase; PLTs, platelets; eGFR, estimated glomerular filtration rate; UACR, urine albumin-creatinine ratio; TG, triglycerides; TC, total cholesterol; LDL, low-density lipoprotein; HDL, high-density lipoprotein; HbA1c, glycated hemoglobin; HOMA-IR, insulin resistance by homeostasis model assessment; T2DM, type 2 diabetes mellitus

Variables	Control group (n=30)	Patients with T2DM (n=60)	P value
Age (years)	48.90±8.15	51.88±9.27	0.09
Sex (male/female)	13/17	27/33	0.1483
BMI (kg/m^2^)	29.84±4.01	37.84±7.08	<0.001*
Waist circumference (cm)	77.3±2.1	95.5±8.21	<0.001*
Systolic blood pressure (mmHg)	116.46±7.41	144.43±20.96	<0.001*
Diastolic blood pressure (mmHg)	77.66±11.57	92.46±15.91	<0.001*
AST (IU/L)	20.3±13.1	22.5±28.6	0.230
ALT (IU/L)	23.06±2.5	26.5±20.67	0.226
Total protein (g/dL)	7.13±0.3	7.1±0.4	0.656
Albumin (g/dL)	4.52±0.1	4.17±0.3	0.065
Total bilirubin (mg/dL)	0.73±0.12	0.8±0.11	0.728
Direct bilirubin (mg/dL)	0.3±13.1	0.4±28.6	0.221
Hemoglobin (g/dL)	13.69±1.68	12.12±2.18	0.311
PLTs (10^6 ^mL/μL)	299.1±101.2	288.14±70.3	0.530
Leukocytes, ×10^9^/L	7.41±2.2	6.14±1.34	0.223
eGFR (mL/minute)	92.37±12.5	71.19±3.2	<0.001*
Serum creatinine (mg/dL)	0.96±0.27	0.97±0.21	0.757
UACR (mg/g)	9.72±2.1	141.97±93.4	<0.001*
Serum urea (mg/dL)	23.3±8.6	24.9±9.18	0.760
TC (mg/dL)	184.9±24.54	224.8±29.68	<0.001*
TG (mg/dL)	150.06±17.18	174.68±26.6	<0.001*
LDL cholesterol (mg/dL)	123.90±21.1	143.6±30.2	<0.001*
HDL cholesterol (mg/dL)	44.1±4.19	39.5±4.41	<0.001*
Fasting plasma glucose (mg/dL)	88.6±7.8	155.7±62.9	<0.001*
Postprandial plasma glucose (mg/dL)	135.0±22.36	260.33±56.0	<0.001*
HbA1c (%)	4.84±0.49	8.30±1.5	<0.001*
Fasting serum insulin (µIU/mL)	6.87±1.91	24.6±13.92	<0.001*
HOMA-IR	1.40±0.438	8.15±5.18	<0.001*
Serum nesfatin-1 level (mmol/L)	9.05±2.10	5.07±1.78	<0.001*

Comparison between diabetes patients with and without microvascular complications

T2DM patients with microvascular complications had a statistically significant higher duration of diabetes, waist circumference, BMI, diastolic and systolic and blood pressure, serum creatinine, UACR, serum urea, TC, TG, LDL cholesterol, fasting plasma glucose (169.97±80.4 versus 128.72±26.9 mg/dL), and postprandial plasma glucose (279.7±110.4 versus 242.3±56.3 mg/dL), while they had statistically significant lower eGFR, HDL cholesterol, and serum nesfatin-1 level (4.32±1.72 versus 5.83±1.51 mmol/L) compared to T2DM patients without microvascular complications. No significant difference was observed in HbA1c, fasting serum insulin, and HOMA-IR (Table 2).

**Table 2:** Comparison between diabetic patients with and without cardiovascular complications according to baseline characteristics and laboratory investigations. *Statistically significant BMI, body mass index; AST, aspartate aminotransferase; ALT, alanine transaminase; PLTs, platelets; eGFR, estimated glomerular filtration rate; UACR, urine albumin-creatinine ratio; TG, triglycerides; TC, total cholesterol; LDL, low-density lipoprotein; HDL, high-density lipoprotein; HbA1c, glycated hemoglobin; HOMA-IR, insulin resistance by homeostasis model assessment

Variables	Diabetic patients without microvascular complications (n=30)	Diabetic patients with microvascular complications (n=30)	P value
Age (years)	52.45±9.16	50.92±9.52	0.782
Sex (male/female)	13/17	12/18	0.500
Duration of diabetes (years)	8.3±5.1	15.5±4.21	<0.001*
BMI (kg/m^2^)	34.18±6.3	38.13±7.96	<0.001*
Waist circumference (cm)	91.3±6.1	98.5±7.21	<0.001*
Systolic blood pressure (mmHg)	137.8±16.4	149.8±20.4	<0.05*
Diastolic blood pressure (mmHg)	86.6±10.5	89.9±11.3	<0.001*
AST (IU/L)	22.3±13.1	22.5±28.6	0.902
ALT (IU/L)	21.06±7.5	27.5±19.67	0.355
Total protein (g/dL)	7.15±0.31	7.06±0.41	0.302
Albumin (g/dL)	4.06±0.1	4.07±0.3	0.848
Total bilirubin (mg/dL)	0.81±0.22	0.81±0.11	0.928
Direct bilirubin (mg/dL)	0.3±13.1	0.4±28.6	0.230
Hemoglobin (g/dL)	12.69±2.68	12.5±1.18	0.808
PLTs (10^6 ^mL/μL)	287.1±67.2	289.14±73.3	0.913
Leukocytes, ×10^9^/L	6.41±1.7	6.8±2.24	0.449
eGFR (mL/minute)	87.37±12.5	59.19±13.2	<0.001*
Serum creatinine (mg/dL)	0.87±0.19	1.09±0.21	<0.001*
UACR (mg/g)	25.72±2.1	191±126.4	<0.001*
Serum urea (mg/dL)	19.3±6.6	25.9±10.18	<0.05*
TC (mg/dL)	206.3±31.90	239.88±22.1	<0.001*
TG (mg/dL)	161.26±22.19	185.16±20.6	<0.001*
LDL cholesterol (mg/dL)	132.08±30.07	151.91±20.4	<0.001*
HDL cholesterol (mg/dL)	41.48±2.87	38.25±4.63	<0.001*
Fasting plasma glucose (mg/dL)	128.72±26.9	169.97±80.4	<0.001*
Postprandial plasma glucose (mg/dL)	242.3±56.3	279.7±110.4	<0.001*
HbA1c (%)	7.83±1.3	8.4±1.6	0.09
Fasting serum insulin (µIU/mL)	21.6±14.2	25.6±13.48	0.129
HOMA-IR	7.5±5.47	8.2±4.18	0.577
Serum nesfatin-1 level (mmol/L)	5.83±1.51	4.32±1.72	<0.001*

Correlation between serum nesfatin-1 level and different baseline characteristics and investigations

There was a strong negative correlation between serum nesfatin-1 level and HOMA-IR and a moderate positive correlation between serum nesfatin-1 level and eGFR and HDL cholesterol, while serum nesfatin-1 level had a moderate negative correlation with the duration of diabetes, waist circumference, TC, TG, serum creatinine, serum urea, and fasting plasma glucose. There exists a weak negative correlation between serum nesfatin-1 level and BMI, LDL cholesterol, and postprandial plasma glucose in patients with T2DM associated with microvascular complications (Table 3).

**Table 3:** Correlation between serum nesfatin-1 level and different parameters in T2DM patients associated with microvascular complications. *Statistically significant BMI, body mass index; eGFR, estimated glomerular filtration rate; UACR, urine albumin-creatinine ratio; TG, triglycerides; TC, total cholesterol; LDL, low-density lipoprotein; HDL, high-density lipoprotein; HbA1c, glycated hemoglobin; HOMA-IR, insulin resistance by homeostasis model assessment

Variables	Serum nesfatin-1 level (mmol/L)	
	R	P
Duration of diabetes (years)	-0.6	<0.001
BMI (kg/m^2^)	-0.268	<0.001
Waist circumference (cm)	-0.562	<0.001
TC (mg/dL)	-0.364	<0.001
TG (mg/dL)	-0.351	<0.001
LDL cholesterol (mg/dL)	-0.268	<0.001
HDL cholesterol (mg/dL)	0.388	<0.001
eGFR (mL/minute)	0.427	<0.001*
Serum creatinine (mg/dL)	-0.391	<0.001*
Serum urea (mg/dL)	-0.314	<0.001*
UACR (mg/g)	-0.022	0.885
Fasting plasma glucose (mg/dL)	-0.330	<0.001
Postprandial plasma glucose (mg/dL)	-0.265	<0.001
HbA1c (%)	-0.229	0.078
HOMA-IR	-0.9	<0.001*

The diagnostic accuracy of serum nesfatin-1 level

**Figure 1:**
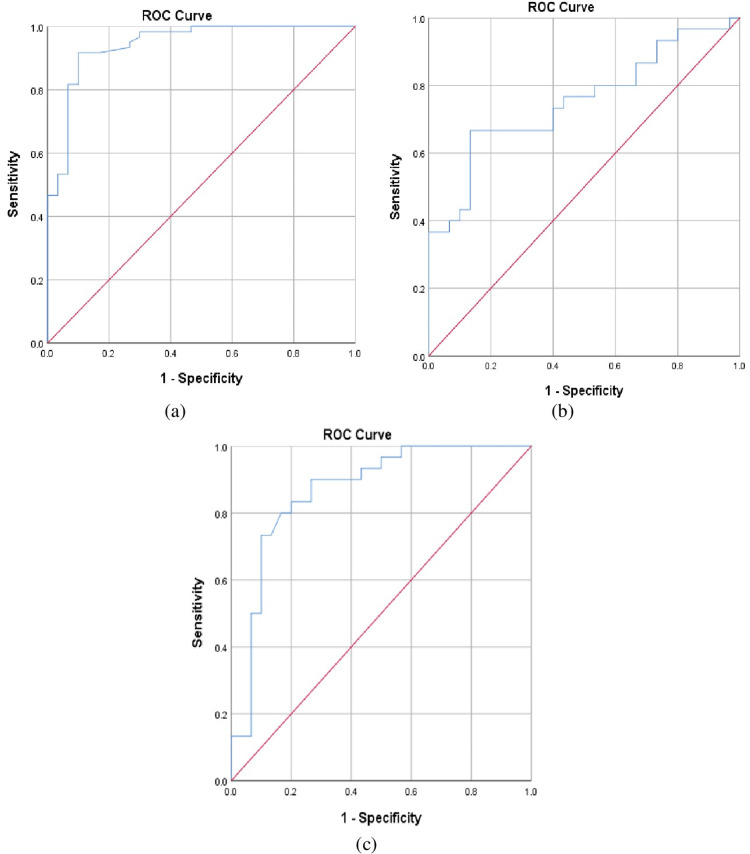
The accuracy of serum nesfatin-1 level for discriminating patients with T2DM from the control group (a), diabetes patients with microvascular complications from patients without microvascular complications (b), and patients with diabetic kidney disease from patients without diabetic kidney disease (c) by ROC analysis. ROC, receiver operating characteristic; T2DM, type 2 diabetes mellitus

Serum nesfatin-1 level at a cutoff value of <8.09 mmol/L can be a marker for the detection of diabetes mellitus with AUC of 94.3%, 95% sensitivity, 74.3% specificity, 77.9% PPV, and 65.7% NPV. Serum nesfatin-1 level at a cutoff value of <5.87 mmol/L can be a marker for the detection of microvascular complications of diabetes mellitus at AUC of 75.5%, 76.7% sensitivity, 67.3% specificity, 77.1% PPV, and 62.9% NPV. Serum nesfatin-1 level at a cutoff value of <5.46 mmol/L could be a marker for the detection of diabetic kidney disease with AUC of 76.1%, 94% sensitivity, 60% specificity, 70% PPV, and 90% NPV (Figure 1).

Multiple stepwise linear regression analyses of patients with microvascular complications

The main independent parameters associated with serum Nesfatin-1 levels are waist circumference and HBA1c (P<0.001, Table 4). 

**Table 4:** Multiple stepwise linear regression analysis was used to test the influence of the main independent variables against serum Nesfatin-1 level (as the dependent variable) in T2DM patients with microvascular complications. CI, confidence interval; SE, standard error

Model		Unstandardized Coefficients		Standardized Coefficients	t	P value	95% CI	
		Beta	SE	Beta			Lower Bound	Upper Bound
1	(Constant)	160.308	17.214		9.313	<0.001	125.850	194.766
	Waist	-0.932	0.180	-0.562	-5.174	< .001>	-1.292	-0.571
2	(Constant)	320.721	7.451		43.043	<0.001	305.800	335.641
	Waist (cm)	-4.273	0.131	-2.577	-32.592	<0.001	-4.535	-4.010
	HBA1c(%)	19.061	0.695	2.167	27.308	<0.001	17.668	20.454

## Discussion

Summary of findings

Our study showed that serum nesfatin-1 level can be a marker for the detection of diabetes mellitus, a marker for the detection of microvascular complications of diabetes mellitus, and a marker for the detection of diabetic kidney disease.

The present study showed that T2DM patients were associated with statistically significant higher waist circumference, BMI, diastolic and systolic blood pressure, UACR, TC, fasting plasma glucose, postprandial plasma glucose, HbA1c, fasting serum insulin, and HOMA-IR, while they had statistically significant lower eGFR, HDL cholesterol, and serum nesfatin-1 level compared to healthy controls.

Comparison with other studies and clinical implication

Numerous research examined the connection between DM risk and anthropometric measures of adiposity. Our research supports the findings of Venkatrao et al.'s study [12], which demonstrated that waist circumference and BMI were both reliable indicators of T2DM risk. Additionally, Gray et al. [13] discovered that obesity and excess weight were significant risk factors for T2DM and its consequences in both males and females. In the overweight category (BMI of 25-29.99), both males and females were more likely to develop diabetes.

As regards blood pressure, Ephraim et al. [14] found in their study on 107 T2DM patients that 37.4% of diabetes mellitus patients had isolated systolic hypertension, which was more common in older adult patients and was linked to elevated renal sodium reabsorption, sympathetic nervous system activation, altered transmembrane ion transport, and the hypertrophy of resistance vessels. On the other hand, a study by Pinto [15] showed that T2DM patients who were 50 or older had an increase in systolic but not in diastolic pressure.

Regarding kidney function, our study agrees with Shahwan et al. [16] who showed that those with T2DM have a high frequency of aberrant renal parameters. Additionally, Ohkuma et al. [17] observed that two-year increases in eGFR and UACR were both highly related to the likelihood of serious medical conditions in their cohort analysis of 8766 T2DM patients. A greater likelihood of T2DM was indicated by both declines in eGFR and rises in UACR over a two-year period, both singly and in conjunction.

Concerning lipid profile, our findings were in line with an earlier investigation by Wang et al. in 2022 [18], which found a favorable correlation between blood glucose levels and TG and LDL-C, which are associated with an increased risk of obesity and cardiovascular disease. Reasonable glycemic management in the context of T2DM may enhance lipid profiles. According to an additional investigation, short-term intensive glycemic management could considerably lower TG levels linked to T2DM [19]. On the other hand, some studies found no association between lipid profile parameters and T2DM [20]. Moreover, a recent study found that fasting plasma glucose is associated with HDL and TC but not with LDL or TG [21].

Regarding serum nesfatin-1 level, our study was in line with previous studies as those conducted by Li et al. [22] and Kadim and Hassan [23], which showed significantly lower nesfatin-1 in the T2DM patients in comparison to the control group. Also, these results were in agreement with the studies done by Matta et al. [24] and Huang et al. [25] who found that nesfatin-1 is lower in both T2DM and prediabetic patients. According to a systematic review and meta-analysis conducted by Zhai et al. [26] for the assessment of circulatory nesfatin-1 levels and T2DM, patients who were just diagnosed with the disease had higher levels of nesfatin-1, while those who were receiving antidiabetic treatment had lower levels. This data raises the possibility that one of the causes underlying diabetic hyperphagia in T2DM patients may be reduced serum levels of nesfatin-1, as nesfatin-1 has been shown to have a lowering influence on food consumption. As a result, it has anorexigenic effects; however, the regulatory process behind this is yet unclear. Additionally, nesfatin-1 affects the control of blood glucose homeostasis, which may explain why T2DM patients lack it [27].

Our study revealed that T2DM patients with microvascular complications had a statistically significant higher duration of diabetes, BMI, waist circumference, systolic and diastolic blood pressure, serum creatinine, UACR, serum urea, TC, TG, LDL cholesterol, fasting plasma glucose, and postprandial plasma glucose, while they had statistically significant lower eGFR, HDL cholesterol, and serum nesfatin-1 level compared to healthy controls.

Nisar et al. [28] emphasized that, in line with our findings, the existence of diabetic neuropathy was highly correlated with HbA1c levels and the length of diabetes. Additionally, Zoungas et al. [29] reported that in individuals who have T2DM, only diabetes-related duration has a separate relationship with microvascular incidents, and this association is stronger in young patients, while age at examination and the length of diabetes have no relationship with macrovascular events and death. Additionally, Cai et al.'s meta-analysis [30] found that greater TG and lower HDL levels may serve as predictors of diabetic neuropathy progression in diabetic individuals. TG and LDL levels that are higher raise the danger of diabetic neuropathy. Al-Ani et al. [31] noted that regardless of the length of the disease, metabolic lipid abnormalities exist alongside neuropathy in T2DM. Additionally, Liu et al. [32] noted a separate relationship between insulin resistance and diabetic cardiovascular autonomic neuropathy (DCAN). It was demonstrated that HOMA-IR is a very precise and economical diagnostic for DCAN screening. Patients at high risk of DCAN had HOMA-IR values of more than 1.735. Additionally, Dai et al. [33] observed that patients with diabetic retinopathy had significantly lower levels of nesfatin-1 than T2DM patients without retinopathy and controls, which is consistent with the current findings. Sun et al. [8] studied the protective function and molecular mechanism of nesfatin-1 against high-glucose-induced inflammatory processes, cellular damage caused by oxidative stress, and death in the retinal epithelial cell [8].

Our results revealed a significant negative correlation between serum nesfatin-1 level and HOMA-IR, a moderate positive correlation between serum nesfatin-1 level and eGFR and HDL cholesterol, and a moderate negative correlation between serum nesfatin-1 level and fasting plasma glucose, waist circumference, TC, TG, serum creatine, and serum urea. In patients with T2DM accompanied by microvascular problems, there is a weak negative connection between serum nesfatin-1 level and BMI, LDL cholesterol, and postprandial plasma glucose. In agreement with our findings, Mohammad and Gallaly [34], Kadim and Hassan [23], and Matta et al. [24] discovered that in diabetes patients, serum nesfatin-1 level had a negative correlation with fasting plasma glucose and HbA1c.

On the other hand, Zhang et al. [35] found that plasma nesfatin-1 correlated positively with BMI, HbA1c, fasting blood glucose, two hours of blood glucose after a glucose load, and fasting plasma insulin; however, Abd-Elaaty et al. [36] and Shaaban et al. [37] showed that no significant correlation was found between plasma nesfatin-1 and HbA1c.

In the current study, the potential diagnostic value of serum Nesfatin-1 level was investigated by ROC curve to distinguish the normal population from T2DM patients, T2DM patients with microvascular complications from those without microvascular complications, and T2DM patients with DKD from those without DKD. We found that serum Nesfatin-1 level at a cut-off value < 8.09 mmol/L could be a marker for the detection of DM with 95% sensitivity and 74.3% specificity, serum Nesfatin-1 level at a cut-off value < 5.87 (mmol/L) could be a marker for detection of microvascular complications with 76.7% sensitivity and 67.3% specificity, and serum Nesfatin-1 level at cut off value < 5.46 (mmol/L) could be a marker for detection of DKD with 94% sensitivity and 60% specificity.

However, the Roc curve analysis in the study done by Matta et al. [24] identified cut-off values of ≤ 9 ng/dl and ≤ 5.5 ng/dl with an AUC of 94% and 0.97, sensitivity of 96.7% and 100%, and specificity of 93.3% and 96.7% for the diagnosis of pre-diabetes and diabetes, respectively. Also, Kadim et al. [23] identified cut-off values of ≤ 29.1 ng/dl with an AUC of 73%, sensitivity of 84%, and specificity of 93.3% for comparing healthy controls and diabetic patients. However, no studies showed the actual normal range of Nesfatin-1 or the difference in cut values due to the different numbers of patients and specific circumstances in each study.

Limitations and recommendations

Some limitations exist in the study such as being a single-center study; therefore, the results can differ in other institutes. Small sample sizes may cause nonsignificant results and the need for the investigation of the role of present comorbidities. Future multicenter studies with a bigger sample size and the investigation of other populations with different ethnic groups are needed to validate our findings and study differences across different populations. Further studies are needed to clarify the relationship between serum nesfatin-1 levels and obesity and whether targeted nesfatin-1 drug therapy can be used as a method of decreasing weight in obese individuals.

## Conclusions

Serum Nesfatin-1 may play a potential protective role in T2DM and its microvascular complications, as it decreases in individuals with diabetes and those with diabetic microvascular complications compared to controls. Additionally, serum Nesfatin-1 levels may have predictive value for the early detection of Type 2 T2DM patients, diabetic microvascular complications, and DKD at cut-off values of < 8.09 mmol/L, < 5.87 mmol/L, and < 5.46 mmol/L, respectively. Therefore, targeted Nesfatin-1 drug therapy may be tried to reduce morbidity and mortality caused by microvascular complications of diabetes.

## References

[1] Fang M, Wang D, Coresh J, Selvin E (2022). Undiagnosed diabetes in U.S. adults: prevalence and trends. Diabetes Care.

[2] Forbes JM, Fotheringham AK (2017). Vascular complications in diabetes: old messages, new thoughts. Diabetologia.

[3] Li H, Lu W, Wang A, Jiang H, Lyu J (2021). Changing epidemiology of chronic kidney disease as a result of type 2 diabetes mellitus from 1990 to 2017: estimates from Global Burden of Disease 2017. J Diabetes Investig.

[4] Wong TY, Cheung CM, Larsen M, Sharma S, Simó R (2016). Diabetic retinopathy. Nat Rev Dis Primers.

[5] Zoungas S, Arima H, Gerstein HC (2017). Effects of intensive glucose control on microvascular outcomes in patients with type 2 diabetes: a meta-analysis of individual participant data from randomised controlled trials. Lancet Diabetes Endocrinol.

[6] Oh-I S, Shimizu H, Satoh T (2006). Identification of nesfatin-1 as a satiety molecule in the hypothalamus. Nature.

[7] Shimizu H, Oh-I S, Hashimoto K (2009). Peripheral administration of nesfatin-1 reduces food intake in mice: the leptin-independent mechanism. Endocrinology.

[8] Sun H, Zhao H, Yan Z, Liu X, Yin P, Zhang J (2021). Protective role and molecular mechanism of action of nesfatin-1 against high glucose-induced inflammation, oxidative stress and apoptosis in retinal epithelial cells. Exp Ther Med.

[9] American Diabetes Association (2022). Standards of medical care in diabetes-2022 abridged for primary care providers. Clin Diabetes.

[10] Sullivan KM, Dean A, Soe MM (2009). OpenEpi: a web-based epidemiologic and statistical calculator for public health. Public Health Rep.

[11] Algul S, Ozkan Y, Ozcelik O (2016). Serum nesfatin-1 levels in patients with different glucose tolerance levels. Physiol Res.

[12] Venkatrao M, Nagarathna R, Majumdar V, Patil SS, Rathi S, Nagendra H (2020). Prevalence of obesity in India and its neurological implications: a multifactor analysis of a nationwide cross-sectional study. Ann Neurosci.

[13] Gray N, Picone G, Sloan F, Yashkin A (2015). Relation between BMI and diabetes mellitus and its complications among US older adults. South Med J.

[14] Ephraim RK, Saasi AR, Anto EO, Adoba P (2016). Determinants of isolated systolic hypertension among diabetic patients visiting the diabetic clinic at the Tamale Teaching Hospital, Northern Ghana. Afr Health Sci.

[15] Pinto E (2007). Blood pressure and ageing. Postgrad Med J.

[16] Shahwan M, Hassan N, Shaheen RA (2021). Diabetes mellitus and renal function: current medical research and opinion. Curr Diabetes Rev.

[17] Ohkuma T, Jun M, Chalmers J (2019). Combination of changes in estimated GFR and albuminuria and the risk of major clinical outcomes. Clin J Am Soc Nephrol.

[18] Wang L, Yan N, Zhang M, Pan R, Dang Y, Niu Y (2022). The association between blood glucose levels and lipids or lipid ratios in type 2 diabetes patients: a cross-sectional study. Front Endocrinol (Lausanne).

[19] Artha IM, Bhargah A, Dharmawan NK (2019). High level of individual lipid profile and lipid ratio as a predictive marker of poor glycemic control in type-2 diabetes mellitus. Vasc Health Risk Manag.

[20] Laverdy OG, Hueb WA, Sprandel MC, Kalil-Filho R, Maranhão RC (2015). Effects of glycemic control upon serum lipids and lipid transfers to HDL in patients with type 2 diabetes mellitus: novel findings in unesterified cholesterol status. Exp Clin Endocrinol Diabetes.

[21] Wang S, Ji X, Zhang Z, Xue F (2020). Relationship between lipid profiles and glycemic control among patients with type 2 diabetes in Qingdao, China. Int J Environ Res Public Health.

[22] Li QC, Wang HY, Chen X, Guan HZ, Jiang ZY (2010). Fasting plasma levels of nesfatin-1 in patients with type 1 and type 2 diabetes mellitus and the nutrient-related fluctuation of nesfatin-1 level in normal humans. Regul Pept.

[23] Kadim BM, Hassan EA (2022). Nesfatin-1 - as a diagnosis regulatory peptide in type 2 diabetes mellitus. J Diabetes Metab Disord.

[24] Matta RA, El-Hini SH, Salama AM, Moaness HM (2022). Serum nesfatin-1 is a biomarker of pre-diabetes and interplays with cardiovascular risk factors. Egypt J Intern Med.

[25] Huang K, Liang Y, Wang K, Wu J, Luo H, Yi B (2022). Influence of circulating nesfatin-1, GSH and SOD on insulin secretion in the development of T2DM. Front Public Health.

[26] Zhai T, Li SZ, Fan XT, Tian Z, Lu XQ, Dong J (2017). Circulating nesfatin-1 levels and type 2 diabetes: a systematic review and meta-analysis. J Diabetes Res.

[27] Mirakhor Samani S, Ghasemi H, Rezaei Bookani K, Shokouhi B (2019). Serum nesfatin-1 level in healthy subjects with weight-related abnormalities and newly diagnosed patients with type 2 diabetes mellitus; a case-control study. Acta Endocrinol (Buchar).

[28] Nisar MU, Asad A, Waqas A (2015). Association of diabetic neuropathy with duration of type 2 diabetes and glycemic control. Cureus.

[29] Zoungas S, Woodward M, Li Q (2014). Impact of age, age at diagnosis and duration of diabetes on the risk of macrovascular and microvascular complications and death in type 2 diabetes. Diabetologia.

[30] Cai Z, Yang Y, Zhang J (2021). A systematic review and meta-analysis of the serum lipid profile in prediction of diabetic neuropathy. Sci Rep.

[31] Al-Ani FS, Al-Nimer MS, Ali FS (2011). Dyslipidemia as a contributory factor in etiopathogenesis of diabetic neuropathy. Indian J Endocrinol Metab.

[32] Liu Y, Peng Y, Jin J (2021). Insulin resistance is independently associated with cardiovascular autonomic neuropathy in type 2 diabetes. J Diabetes Investig.

[33] Dai R, Deng G, Sun Z, Liu Z, Qian Y, Han Y (2017). Relation of serum and vitreous nesfatin-1 concentrations with diabetic retinopathy. J Clin Lab Anal.

[34] Mohammad N, Gallaly DQ (2020). Serum Nesfatin-1 in patients with type 2 diabetes mellitus: a cross sectional study. Zanco J Med Sci.

[35] Zhang Z, Li L, Yang M, Liu H, Boden G, Yang G (2012). Increased plasma levels of nesfatin-1 in patients with newly diagnosed type 2 diabetes mellitus. Exp Clin Endocrinol Diabetes.

[36] Abd-Elaaty T, Rezk MM, Moneium HH, Naga YS, Ghoniem SS (2017). Study of the association of serum level of nesfatin-1 and diabetic kidney disease in patients with type 2 diabetes. Egypt J Obes Diabetes Endocrinol.

[37] Shaaban MA, Dawood AA, Tawfik AR, Nooh MZ, Montaser BA, Elnagdy ME (2021). The relationship between nesfatin-1 and grades of diabetic nephropathy in type 2 diabetic patients. Menouf Med J.

